# Averting expenditure on malaria: effects on labour productivity of maize farmers in Bunkpurugu-Nakpanduri District of Ghana

**DOI:** 10.1186/s12936-020-03521-0

**Published:** 2020-12-03

**Authors:** Franklin Nantui Mabe, Thomas Dafurika

**Affiliations:** Department of Agricultural and Resource Economics. Faculty of Agribusiness and Applied Economics, University for Development Studies, Nyankpala Campus, Tamale, Ghana

**Keywords:** Averting expenditure, Malaria, Labour productivity, Conditional mixed process

## Abstract

**Background:**

Malaria has been one of the commonest diseases during farming season, which affects farmers’ health resulting in a reduction in the number of days spent on the farm. As a result, farmers are regularly trying to avert malaria infection through preventive measures. Motivated by this argument, this study sought to determine the effects of malaria averting expenditure on labour productivity of maize farmers in Bunkpurugu-Nakpanduri District in the Northern Region of Ghana.

**Methods:**

A cross-sectional primary data was collected from 194 maize farmers. Both descriptive and quantitative data analysis approaches were employed. Conditional mixed process was used to estimate the effects of malaria prevention expenditure on maize farmers’ labour productivity.

**Results:**

The study revealed that maize farmers incurred an average expenditure of GHc284.6 to prevent malaria annually. The result shows that factors that affect maize farmers’ malaria prevention expenditure include off-farm income, household size, presence of bushes around houses, presence of pregnant women and number of household members in school. Meanwhile, quantity of fertilizer, seed, weedicides, farming experience, age, ownership of motorbike and averting expenditure are significant determinants of maize labour productivity. The study revealed that farmers who spend more money to avert malaria attack are more labour productive.

**Conclusions:**

Therefore, this study recommends that Ministry of Health and Ministry of Food and Agriculture should collaborate and integrate health extension service on malaria in agricultural extension to educate farmers on the need to avert malaria. Farmers should be educated on malaria preventive strategies, such as clearing of bushes around houses, draining of stagnant water, sleeping in treated mosquito nets among others. Lastly, aside distribution of free mosquito nets to pregnant women, they should be subsidized and made available to all farmers for malaria prevention.

## Background

Malaria is prevalent and perennial in all parts of Ghana with seasonal variations that are more severe in the Northern part of Ghana. The duration of malaria transmission season in Ghana varies from one geographical region to another. This depends on the length of the dry season during which there is a little transmission. In the Northern part of the country, there is a 6–7 month transmission season with the highest number (50–60%) of cases occurring between July and November [1].

Research has revealed that malaria imposes significant burden on the economic growth of developing countries. The effects of malaria on adults are very serious. In addition to the time and money spent on preventing and treating malaria, it causes considerable pain and weakness among the afflicted. These, therefore, reduce people’s working capacity and hence decrease productivity. Ghana as a country is implementing a strategic action plan aimed at reducing the burden of malaria by 75% [2]. To be able to achieve this, the general population especially farmers who are much exposed to the vector need to take centre stage. Farmers are mostly exposed to mosquitoes due to the nature of their work. Mosquitoes are in abundance during the raining season, when farming activities are more pronounced. Farming is done in bushes where mosquitoes rest and breed. Also, due to the nature of farmers’ work, they are sometimes compelled to cross rivers, streams and ponds which are breeding places for the vectors of the parasites that cause malaria. Above all, farming is done by rural households who stay in less mosquito-proof houses.

The above-mentioned predicaments of farmers are not different from what pertains in Bunkpurugu-Nakpanduri District. Malaria affects farmers and their children thereby resulting in substantial economic losses because of the days they have to spend at home or in hospitals treating the disease. Labour is lost due to farmers’ inability to work when attacked by malaria. According to Snowden [3], health expenditures on malaria drain limited resources of households. Snowden also estimated that when malaria prevalence is reduced, long term productivity would be improved substantially.

Household expenditures on malaria can be put into two main components; expenditure on averting and cost on malaria related treatment which include direct payment for drugs, consultation, laboratory test, transport fees to and from healthcare providers and cost of subsistence at distance health facilities [4]. The expenditure on malaria that is incurred by the farmers in the Northern Region of Ghana is a major challenge that hinders the economic development in the Region and the country at large. Snowden [3] explicitly said that “treating malaria is very expensive, preventing malaria is very cheap”. This assertion corroborates the adage that “prevention is better than cure”.

Malaria makes people feel weak and have general malaise hence their inability to work. Farmers are most likely to lose more on this because of the strenuous nature of their work. A study by Fink and Masive [5] revealed that farmers who were given bed nets were able to increase their harvest value by 15%. This suggests that if farmers spend money on bed nets to avert the occurrence of malaria, they are likely to increase the revenue they will obtain from farming by 15%. This might only be beneficial if the money spent on bed nets is less than the 15% revenue obtained for averting malaria. Many researchers have looked at the impact of malaria on agricultural productivity. In a study on the economic benefits of malaria eradication, Snowden [3] concluded that malaria renders people less productive in the short-term, but the long-term level of the impact is highly unpredictable.

According to Asenso-Okyere and Dzator [4], on the average, 3 work days are lost per fever episode by the patient and 2 work days by the caretaker. The monetary value of these lost work days to the management and treatment of fever per episode is USD 6.87 and this formed about 79 percent of the cost of seeking treatment in 1994. Research on whether or not averting cost expenditure improves labour productivity is limited. Though several researches have been conducted to get the cost involved in treating and preventing malaria, there is still a wide gap to be filled. The annual averting expenditure on malaria among maize farmers in the Ghanaian context is a gap needed to be filled. Also, the socio-economic determinants of averting expenditure on malaria among farmers have not been given the needed attention. In addition, whether averting improves labour productivity or not is another bone of contention which needs to be unraveled. The effects of malaria on farmers in the Bunkpurugu-Nakpanduri District is a major concern which needs to be given attention. The main occupation of the majority in the district is farming. This implies that the livelihood of a household will be threatened when there is incidence of malaria because it will largely affect labour productivity. Therefore, this study sought to estimate annual malaria averting expenditure that maize farmers incur and determine whether or not the averting expenditure improves labour productivity in the Bunkpurugu-Nakpanduri District of Ghana.

### Effects of malaria on agricultural labour productivity

Malaria averting expenditure is the cost one incurs in trying to avoid or prevent malaria. Studies have revealed that an annual investment target per person at risk will be US$3.90 by 2020, US$4.30 by 2025 and US$4.4 by 2030 [6]. This, therefore, suggests that on average, an annual amount of not less than US$3.00 will be required to spend on each person towards prevention and elimination of malaria.

The annual cases of malaria reports in Ghana are very alarming and have been a major concern to various stakeholders. In response to this, an average amount of US$20 million is spent annually to get rid of it. In 2018 fiscal year, an amount of US$26 million was invested for malaria prevention and control [7].

The estimated cost of malaria to businesses in Ghana in 2014 was about US$658 million [1].

The incidence of malaria disease has a lot of significant effects on agricultural productivity as it leads to loss of productive time by both the affected person and the caretaker. A study conducted in Nigeria has revealed that the direct economic cost comprises the effect of malaria caused by mortality, morbidity and disability on individual, household and national labour supply, productivity and output [8]. Poor health would lead to hardship on farming and households including monetary expenditures, loss of labour days, and sometimes death. Another study has shown that fishermen who were affected by malaria had lower productivity due to the number of days lost from the malaria infection [9]. Households that are inflicted with malaria are less productive than households who are not inflicted with malaria [10]. According to Asiamah et al*.* [11], farmers who are affected by malaria normally end up ploughing, sowing, weeding, harvesting late among other farm activities, which results in low productivity.

## Methods

### Study area

Bunkpurugu-Yunyoo District now Bunkpurugu-Nakpanduri District is one of the under-developed Districts in the North East Region of Ghana. According to the 2010 population and housing census, the district has a population of about 122,591 with males and females constituting 49.1% and 50.9%, respectively [12]. The District lies in the tropical continental belt with climate conditions suitable for mosquitoes. The percentage of households in agriculture is about the same for the rural (98%) and urban (97.9%) localities. Most households in the District (97.9%) are involved in crop farming [12].

### Sampling procedure and data collection methods

The study used primary data, which were collected using a semi-structured questionnaire. Simple random sampling technique was used to select 10 communities with 20 respondents for each community. This gave a total sample size of 200 respondents for the study. The data were entered into SPSS version 25 and the analysis was done using excel and Stata version 15.

### Theoretical framework of averting expenditure on malaria

The theoretical framework of this study is backed by defensive behaviour or averting behaviour theory. It is a revealed preference approach based on a health production function which was first propounded by Grossman [13] with an extension to the model by Harrington and Portney [14]. The model is based on the ideology that an individual experiences some health output, such as a number of days spent on sick bed and this enters into his/her utility function causing disutility [15]. To avoid this disutility, the individual tries to avert.

According to Sonia and Khawaja [16], averting behaviour models take into account any action or expenditures that individuals undertake to avoid an illness. Averting behaviour models are preventive expenditures or actions that individuals undertake to avoid exposure to any undesirable outcome. This is an approach that says that the value of a small reduction in health state can in principle be measured by the amount an individual is willing to spend on some defensive or averting action to prevent it.

The assumption in the averting behaviour approach in this study is that farmers make choices in order to spend in preventing malaria, which will help to maximize their crop labour productivity in the long-run. Farmers’ crop labour productivity may be influenced by various factors, such as farm size, age, education, weedicides used, pesticides used, improved seeds used, and finally averting action taken by the individual to reduce the experiences as a negative health outcome.

### Models and method of data analysis

#### Simple arithmetic summation

Simple arithmetic summation was used to estimate the annual averting expenditure for malaria. This was done by multiplying a quantity of the items used by the household in averting malaria with their respective prices. After getting the amount spent on each method of averting, their total summation was computed to obtain the total annual averting cost as shown below.

1 $$\begin{aligned} AEM_{i} &= P_{{MN_{i} }} Q_{{MN_{{_{i} }} }} + P_{{MC_{i} }} Q_{{MC_{i} }} + P_{{AR_{i} }} Q_{{AR_{i} }} + P_{{MR_{i} }} Q_{{MR_{i} }} \\ & \quad + P_{{NW_{i} }} Q_{{NW_{i} }} + P_{{SD_{i} }} Q_{{SD_{i} }} + P_{{PC_{i} }} Q_{{PC_{i} }} + P_{{AT{}_{i}}} Q_{{AT_{i} }} \\ \end{aligned}$$

where $AEM$ is the annual averting expenditure on malaria; $P_{MN}$ is the price of mosquito nets; $Q_{MN}$ is the number of mosquito nets used by the household per year; $P_{MC}$ is the price of mosquito coils; $Q_{MC}$ is the quantity of mosquito coils used by the household per year; $P_{MR}$ is the price of mosquito repellents; $Q_{MR}$ is the quantity of mosquito repellents used by the household per year; $P_{AR}$ is the price of aerosol spray; $Q_{AR}$ is the quantity of aerosol spray used by the household per year; $P_{NW}$ is the price of weeding the compound of household; $Q_{NW}$ is the number of times a household compound is weeded per year; $P_{SD}$ is the Price of draining off stagnant water; $Q_{SD}$ is the number of times stagnant water is drained off by the household per year; $P_{PC}$ is the price of protective clothing; $Q_{PC}$ is the number of protective clothing’s used by the household per year; $P_{AT}$ is the price of anti-malarial tablets; $Q_{AT}$ is the quantity of anti-malarial tablets used by the household per year. Note that all the prices are in Ghana cedis (Gh¢).

#### Conditional recursive mixed process

Conditional recursive mixed process (CMP) is a system of equations, which estimates recursive effects of variables on the overall outcome variable. To estimate the impact of malaria averting expenditure on maize labour productivity, one needs to estimate two sets of equations. The first equation deals with factors influencing amount of money farmers spend to avert malaria attack. The second equation looks at the effects of malaria averting expenditure on maize labour productivity of farmers. Malaria averting expenditure is an endogenous variable and failure on the part of a researcher to account for the endogeneity may lead to inconsistent estimates. Also, self-selection of some farmers to avert more as compared to others due to certain characteristics that distinguish them can result in sample selection bias. With such a problem, the real impacts of malaria averting expenditure on maize labour productivity may be exaggerated. To deal with this sample selection bias and endogeneity without going through the problem of finding a good instrument, CMP developed by Roodman [17] is used to jointly estimate the two equations.

CMP follows the concept of seemingly unrelated regression, which assumes that there are high levels of independence among different systems of equations [17–19]. It has the ability to deal with incidence of endogeneity [18, 19]. In this study, a CMP with Ordinary Least Square (OLS) Estimator was used. The first equation of CMP which looks at factors influencing the amount of money farmers spend to avert the incidence malaria is given as:

2 $$\begin{aligned} AEM_{i} & = \beta_{0} + \beta_{1} Sex_{i} + \beta_{2} HHS_{i} + \beta_{3} Age_{i} + \beta_{4} Edu_{i} + \beta_{5} Bush_{i} \\ & \quad + \beta_{6} Stg\_wat_{i} + \beta_{7} Pg\_wmn_{i} + \beta_{8} HH\_edu_{i} + \beta_{9} Off\_Inc_{i} + \mu_{i} \\ \end{aligned}$$

To estimate the effects of averting expenditure on maize labour productivity, the outcome model which is the second equation of CMP is stated as:

3 $$\begin{aligned} LP_{i} & = \lambda_{0} + \lambda_{1} Cap_{i} + \lambda_{2} Fert_{i} + \lambda_{3} Seed_{i} + \lambda_{4} Wd_{i} + \lambda_{5} Fs_{i} + \lambda_{6} Exp_{i} \, + \lambda_{7} Ext_{i} + \lambda_{8} Sex_{i} \\ & \quad + \lambda_{9} HHS_{i} + \lambda_{10} Age_{i} + \lambda_{11} Edu_{i} + \lambda_{12} Mot_{i} + \lambda_{13} AEM_{i} + \lambda_{14} HH\_Edu_{i} + \varepsilon_{i} \\ \end{aligned}$$

*β* and *λ* are coefficients which measure the effects of the explanatory variables on the malaria averting expenditure and labour productivity respectively. The definition and measurement of the variables in Eqs. (2) and (3) are illustrated in Table 1 under “Summary statistics of variables” section.

**Table 1:** Summary statistics of variables

Variables	Definitions and measurements	Mean	Std. deviation	Min	Max
LP	Labour productivity (Mt/man-day)	0.09	0.08	0.00	0.54
Cap	Capital (Gh¢)	206.73	116.41	59.67	941.33
Fert	Fertilizer (kg)	396.13	275.79	100.00	1600.00
Seed	Seeds (kg)	88.84	59.57	15.00	459.00
Wd	Weedicides (L)	4.08	4.70	0.00	25.00
Fs	Farm size (ha)	1.18	0.62	0.40	4.05
Exp	Farming experience (years)	38.12	13.84	3.00	75.00
Ext	Number of extension officers visits	0.67	1.23	0.00	5.00
Sex	Sex (male = 1, female = 0)	0.53	0.50	0.00	1.00
HHS	Household size	7.86	2.53	2.00	16.00
Age	Age (years)	51.28	13.56	25.00	86.00
Edu	Educational status of household head (educated)	0.56	0.50	0.00	1.00
Mot	Ownership of motor bike (1 = yes, 0 = no)	0.40	0.49	0.00	1.00
AEM	Averting expenditure for malaria (Gh¢)	284.60	133.07	24.00	990.00
Bush	Presence of bushes around the house (1 = yes, 0 = no)	0.64	0.48	0	1
Stg_wat	Presence of stagnant water (1 = yes, 0 = no)	0.67	0.47	0	1
Pg_wmn	Presence of pregnant woman (1 = yes, 0 = no)	0.18	0.39	0	1
HH_edu	Household members in education	3.97	1.99	0	9
Off_Inc	Off-farm income of household (Gh¢)	1650.48	1313.76	234.00	7495.00

Labour productivity (LP) is a partial productivity measurement, which is estimated as the quantity and quality of output per unit labour. As output is measured in metric tons and labour in man-days, the unit of measurement of labour productivity is metric tons per man-day.

## Results and discussion

### Summary statistics of variables

Table 1 depicts the summary statistics of the variables used in the CMP model. As shown in the table, the average maize labour productivity is 0.09 Mt/man-day whereas the maximum productivity obtained by a farmer was 0.54 Mt/man-day. On average, a farmer spends Gh¢2016.73 on fixed inputs, 396.1 kg of fertilizer, 88.8 kg of maize seed and 4.1 L of weedicides to cultivate 1.2 ha of maize. This finding underscores the fact that most rural farmers are still engaged in subsistence level farming which confirms the findings of Ibrahim and Shaibu [8]. Whilst the average age of a farmer is 51.3 years, the average farming experience was 38.1 years. Hence, the average of the workforce in farming in the study area is very high. This implies that farmers in Ghana are ageing. This can be attributed to the fact that the youth do not see farming as an attractive occupation. The reason can also be that most of the youth have received some level of formal education and hence migrate to cities in search for ‘non-existing’ white-collar jobs. The mean household size is 7.9 which means that farm households will often depend on the workforce of the family labour for their farming activities. This is in line with Ibrahim and Shaibu [8], who state that farm households depend on a pool of family labour for farm operations.

This could be due to the fact that the youth of today are not interested in farming. The number of extension visits per farmer is very low thus 0.7 times per annum. Out of 194 farmers interviewed, 53% were males. Though women are noted to be largely engaged in farming than men in Northern Ghana they do not have access and control over land due to the nature of the land tenure system. This confirms the findings of Fon [20] that a majority (75.7%) of women are believed not to have access and control over land. This is one of the reasons why the percentage of women engaged in maize farming in the study area is low.

The percentage of household heads with at least primary school education was 56%. The sanitation and hygiene are poorly observed by the respondents with 64% and 67% having bushes and stagnant water around their houses respectively. This suggests that most farmers will spend more in clearing the bush to reduce breeding of mosquitoes, which can raise their averting expenditure.

Simple arithmetic summation was used to estimate the averting expenditure among maize farmers in the Bunkpurugu-Nakpanduri District. As depicted in Table 1, the mean annual malaria averting expenditure is GH¢284.6. The results corroborated with the findings of Gunda et al*.* [21] that household’s expenditure on malaria is GH¢554.40 (for both prevention and treatment). The minimum and maximum expenditures of households on preventing malaria are GH¢24.00 and GH¢990.00 respectively. The vast difference between the minimum and the maximum averting expenditure is attributed to some of the demographic characteristic differences such as bush, stagnant water around houses, household size, income among others.

### Determinants of averting expenditure on malaria and labour productivity

In order to identify factors influencing malaria averting expenditure as well as maize production labour productivity, CMP framework with ordinary least square regression model was used (see Table 2). This was done to solve the problem of endogeneity of averting expenditure variable. The *atanhrho_12* is negative but insignificant. This implies that there are no unobserved factors affecting malaria averting expenditure as well as maize production labour productivity. Hence there is no selectivity bias. The 1% significance of the likelihood ratio test suggests that there is a correlation between the error terms of the malaria expenditure and the maize labour productivity models. Therefore, the two models could not have been estimated individually [22].

**Table 2:** Determinants of and effects of malaria averting expenditure on maize labour productivity. Source: Analysis from field data (2019)

Variables	Coef.	Std. error
First model: averting expenditure		
Sex	7.05682	17.98260
HHS	7.32490*	4.11162
Age	0.04406	0.68027
Edu	30.24928	18.44907
Bush	50.67986***	18.53492
Stg_wat	− 5.69713	18.19524
Pg_wmn	45.18058**	22.64726
HH_edu	11.20739**	5.02365
Off_inc	0.01444**	0.00657
_cons	98.85385	48.11921
Second model: maize labour productivity		
Cap	− 0.000074	0.000179
Fert	0.000055*	0.000030
Seed	0.000730**	0.000359
Wd	0.002393**	0.000984
FS	− 0.016151	0.012121
Exp	− 0.001852***	0.000610
Ext	− 0.004596	0.003355
Sex	0.001231	0.008826
HHS	− 0.000584	0.002404
Age	0.001614**	0.000630
Edu	0.001935	0.010474
Mot	0.017812*	0.009248
AEM	0.000239*	0.000128
HH_edu	− 0.004548	0.002910
_cons	− 0.038118	0.029423
/lnsig_1	4.772509***	0.050808
/lnsig_2	− 2.855080***	0.090131
/atanhrho_12	− 0.291986	0.274319
sig_1	118.2154	6.006326
sig_2	0.057551**	0.005187
rho_12	− 0.283962	0.252199
Number of obs	194	
LR chi^2^ (23)	157.03	
Log likelihood	− 914.37459***	
Prob > chi^2^	0.0000	

^***^, ** and ** are significant at 1%, 5% and 10% respectively

### Socio-economic determinants of averting expenditure on malaria

Results from the analysis showed that off-farm income, presence of pregnant women in a household and number of children in school are statistically significant at 5% each. Whilst presence of bushes around the houses was statistically significant at 1%, household size was statistically significant at 10%. This suggests that off-farm income, household size, number of children in school, presence of pregnant women and bushes around the houses are significant determinants of averting expenditure on malaria.

The coefficient of off-farm income implies that if a household’s off-farm income increases by GH¢1.00, the amount of money spent by the household in averting malaria will increase by GH¢0.014 ceteris paribus (holding other factors constant). This is in line with the findings of McElroy et al*.* [23] that, household level of expenditure in preventing malaria will increase as their income level increases.

Also, the coefficient of household size is 7.32 which means that if one person is added to the household, their averting expenditure will increase by GH¢7.32 all other things being equal. This makes economic sense because as household size increases, their averting materials will equally increase as well and hence the results. In addition, the coefficient of households with bushes is 50.68. This implies that households that are having bushes around their houses are more likely to spend GH¢50.68 more than their counterparts ceteris paribus. This is because bushes breed mosquitoes and households will put more effort to get rid of it and hence spending more to avert than houses without bushes. The findings of this study corroborate with the work of Mbako et al*.* [24] that clearing bushes around houses sustainably empowers and strengthens rural communities to reduce mosquito breeding sites. The clearing of bushes around houses require financial expenditure hence the positive direction of its effects of annual malaria averting expenditure.

The coefficient of presence of pregnant woman or women in a household is 45.18. This suggests that households with pregnant women are more likely to spend GH¢45.18 more than households without pregnant women. This confirms the findings of Sabin et al*.* [25] that most pregnant women are willing to try new methods of malaria prevention although cost related barriers to such methods were stressed. This is because pregnant women are more susceptible to malaria, and hence households need to employ all means to get rid of mosquitoes and as a result spend more to avert than households without pregnant women. Lastly, if the number of school children in a household increases by one, the amount of money the household will spend to avert malaria will increase by GH¢11.21. School children sometimes educate the parents about the importance of malaria averting as compared to prevention.

### Effects of malaria averting expenditure on maize labour productivity

The results show that malaria averting expenditure, farming experience, age of household head, and ownership of motorbike were the significant socio-economic factors that influence labour productivity.

The coefficient of malaria averting expenditure suggests that if farmers increase their malaria averting expenditure by GH¢1.00, their maize labour productivity will increase by 0.024% Mt/man-day ceteris paribus. Malaria is the common disease during farming season, which has a lot of effects on farm labour productivity. Farmers who avert malaria are at less risk to malaria infection and are likely to be more productive as compared to their counterparts who did not avert malaria. This is based on the positive relation of malaria averting expenditure with maize labour productivity which agrees with the findings of Kioko [10] that households afflicted with malaria have lower crop output compared with households that are not afflicted with malaria.

Farming experience variable suggests that as number of years of farming increases by one, the maize labour productivity of the farmer decreases by 0.19% Mt/man-day ceteris paribus. Experience is an important factor in determining labour productivity and as a result of that, its contribution cannot be overlooked. This might be that farmers who are relatively older may be less productive in maize farming. As farmers’ advance in age, they become weak and are no more effective as they were young and hence the negative relationship of farming experience with labour productivity. This agrees with the findings of Guo et al*.* [26] that an increase in age is not conducive to improving agricultural output. This is at variance with the findings of Afari [27] that farmers who are more productive may have spent a greater part of their formative years on the farm and have at least learnt a lot of skills (at least in traditional way) in making good use of available inputs at their disposal. The co-efficient of age implies that the labour productivity of farmers will increase by 0.16% Mt/man-day for a 1-year increase in the age. Also, farmers who own motorbikes are relatively highly productive than their counterparts. As shown in Table 2, a farmer who owns a motorbike is 1.78% more labour productive than his or her counterpart. This is because a motor bike provides a faster means of transport to farmers thereby sparing them more time for farm work. With motorbikes, the energy that could have been used to pedal bicycle or walk to the farm is spared for working on the maize farm.

For production variables, whilst the level of significance of quantity of fertilizer is 1%, seed, and weedicides are statistically significant at 5% each. A 1 kg increase in the quantity of fertilizer applied will increase maize labour productivity by 0.01% Mt/man-day. Similarly, maize labour productivity increases with quantity of weedicides and seeds used.

### Farmers perceive benefit of averting expenditure on malaria

Table 3 depicts the percentage distribution of farmers’ responses on perceived benefits of averting malaria. From the results, farmers perceived that spending money or energy to prevent the malaria helps to increase the number of times one goes to farm. Out of 194 respondents, 59.5% strongly agreed to this. Whilst 43.5% and 51.5% of the respondent respectively strongly agreed and agreed that when one spends money to avert malaria infection, he/she saves income. This is because the person would not have to spend money to cure himself or herself from malaria. Most of the farmers were of the view that when they avert malaria, they will save their income for other purposes.

**Table 3:** Percentages of the perceived benefits of averting malaria. Source: Analysis from field (2019)

Variable	Strongly agree	Agree	Neutral	Disagree	Strongly disagree
Averting helps me increase the number of times I go to farm	59.50	38.00	2.50	0.00	0.00
Averting malaria help you not to spend in treating malaria	35.50	62.50	2.00	0.00	0.00
Averting malaria will help me save income	43.50	51.50	5.00	0.00	0.00
Averting malaria will help me increase my labour productivity	46.00	50.50	2.00	1.00	0.50
Averting malaria will make me be healthy and active	51.50	41.50	2.00	0.00	1.00
Averting malaria will help reduce my health expenditure	48.00	49.00	2.50	0.00	0.50
Averting malaria will help me avert other diseases	48.00	47.50	3.50	1.00	0.00
Occurrences of sickness will reduce in my house if I avert malaria	48.50	48.00	2.00	1.00	0.50

In addition, averting malaria helps one to increase labour productivity. As shown in Table 3, a total of 96.5% of the farmers interviewed perceived that averting of malaria keeps them healthy to work in the farm and get higher output. This is corroborated by the perception that averting prevent them from spending money to treat malaria. Farmers’ explanation was that one can only spend money in treating malaria if the person is infected with malaria but if one averts malaria, one will be free from malaria attack and hence does not need to spend money on that. The general body weakness caused due to malaria infection is undisputable and it always makes the victim inactive over time. Other important benefits of spending money or energy to avert malaria infection include good health and prevention from other diseases. Malaria is the common disease that affects farmers during the farming season. This is the time most hospitals record high malaria cases.

## Conclusions

The research has revealed that all the farmers interviewed expend some money and effort to avert malaria. They use one or more malaria averting strategies. This can be inferred from household average malaria averting expenditure of GH¢284.60. This is further confirmed by the farmers perceived benefit of averting malaria with more than 90% of the respondents strongly agreeing and agreeing with the various perceived benefits. The research has also discovered that averting expenditure on malaria leads to an increase in maize labour productivity. Malaria averting expenditure means that farmers will be free from malaria infection and, therefore, the number of days lost due to ill health will reduce and hence lead to an increase in their labour productivity.

It is recommended that Ministry of Health and Ministry of Food and Agriculture should collaborate and integrate health extension service on malaria into agricultural extension to educate farmers on the need to avert malaria. Farmers should be educated on malaria preventive strategies, such as clearing of bushes around houses, draining of stagnant water, sleeping in treated mosquito nets among others. Also, aside distribution of free mosquito nets to pregnant women, they should be subsidized and made available for all farmers to procure for malaria prevention.

## Data Availability

The data is available and will be provided upon official request.

## Abbreviations

AEM: Averting expenditure on malaria
CMP: Conditional recursive mixed process
